# Synergistic Effect of Melatonin and *Lysinibacillus fusiformis* L. (PLT16) to Mitigate Drought Stress via Regulation of Hormonal, Antioxidants System, and Physio-Molecular Responses in Soybean Plants

**DOI:** 10.3390/ijms24108489

**Published:** 2023-05-09

**Authors:** Muhammad Imran, Clems Luzolo Mpovo, Muhammad Aaqil Khan, Shifa Shaffique, Daniel Ninson, Saqib Bilal, Murtaza Khan, Eun-Hae Kwon, Sang-Mo Kang, Byung-Wook Yun, In-Jung Lee

**Affiliations:** 1 Biosafety Division, National Institute of Agriculture Science, Rural Development Administration, Jeonju 54874, Republic of Korea; m.imran02@yahoo.com; 2 Department of Applied Biosciences, Kyungpook National University, Daegu 41566, Republic of Korea; 3 Department of Chemical and Life Sciences, Qurtuba University of Science and Information Technology, Peshawar 24830, Pakistan; 4 Natural & Medical Sciences Research Center, University of Nizwa, Nizwa 616, Oman; 5 Department of Horticulture and Life Science, Yeungnam University, Gyeongsan 38541, Republic of Korea

**Keywords:** melatonin, plant-growth-promoting rhizobacteria (PGPR), soybean, drought, antioxidants, phytohormones

## Abstract

Drought is one of the most detrimental factors that causes significant effects on crop development and yield. However, the negative effects of drought stress may be alleviated with the aid of exogenous melatonin (MET) and the use of plant-growth-promoting bacteria (PGPB). The present investigation aimed to validate the effects of co-inoculation of MET and Lysinibacillus fusiformis on hormonal, antioxidant, and physio-molecular regulation in soybean plants to reduce the effects of drought stress. Therefore, ten randomly selected isolates were subjected to various plant-growth-promoting rhizobacteria (PGPR) traits and a polyethylene-glycol (PEG)-resistance test. Among these, PLT16 tested positive for the production of exopolysaccharide (EPS), siderophore, and indole-3-acetic acid (IAA), along with higher PEG tolerance, in vitro IAA, and organic-acid production. Therefore, PLT16 was further used in combination with MET to visualize the role in drought-stress mitigation in soybean plant. Furthermore, drought stress significantly damages photosynthesis, enhances ROS production, and reduces water stats, hormonal signaling and antioxidant enzymes, and plant growth and development. However, the co-application of MET and PLT16 enhanced plant growth and development and improved photosynthesis pigments (chlorophyll a and b and carotenoids) under both normal conditions and drought stress. This may be because hydrogen-peroxide (H_2_O_2_), superoxide-anion (O^2−^), and malondialdehyde (MDA) levels were reduced and antioxidant activities were enhanced to maintain redox homeostasis and reduce the abscisic-acid (ABA) level and its biosynthesis gene *NCED3* while improving the synthesis of jasmonic acid (JA) and salicylic acid (SA) to mitigate drought stress and balance the stomata activity to maintain the relative water states. This may be possible due to a significant increase in endo-melatonin content, regulation of organic acids, and enhancement of nutrient uptake (calcium, potassium, and magnesium) by co-inoculated PLT16 and MET under normal conditions and drought stress. In addition, co-inoculated PLT16 and MET modulated the relative expression of *DREB2* and TFs *bZIP* while enhancing the expression level of *ERD1* under drought stress. In conclusion, the current study found that the combined application of melatonin and Lysinibacillus fusiformis inoculation increased plant growth and could be used to regulate plant function during drought stress as an eco-friendly and low-cost approach.

## 1. Introduction

Plants are sessile organisms; hence, they must cope with harsh environmental conditions in order to avoid negative consequences for their growth and development [1,2]. Drought, cold, heat, salt, ultraviolet rays, and waterlogging are all becoming more common as a result of global warming and climate change, posing serious concerns for crop output, sustainable agriculture, and global food security [2]. Among these environmental impacts, drought is considered an intricate and prolonged phenomenon that negatively affects agriculture, the economy, water supplies, and ecosystems [3]. Furthermore, it can be determined as a lack of water such as precipitation, soil moisture, or streamflow in each water cycle [4]. In addition, due to drought, alterations in the colloidal–chemical composition of the cytoplasm result in a lack of water, protein breakdown, and a reduction in the number of organic compounds collected in plants, which lead to osmotic stress induced by a decrease in water potential caused by high temperatures and dryness [5]. Drought-induced stress damages and weakens cell macromolecules, causing peroxidation of membrane lipids, inactivating enzymes, and inhibiting the cell cycle [6,7]. The response of plants to stress is determined by the severity and length of the stress. Drought creates an overabundance of reactive oxygen species (ROS), which causes antioxidant enzymes such as superoxide dismutase (SOD), catalase (CAT), ascorbate peroxidase (APX), and glutathione (GSH) to be activated [2].

The impacts of drought on soybean have been widely documented. Drought causes morphological alterations in the vegetative plant, as well as reduced photosynthesis and water status in plant leaves and peroxidation of the membrane lipids. Substantial efforts have been made to improve soybeans’ ability to withstand drought, with the main objective being to increase production under stress [8,9]. Given that soybeans are one of the ten most widely grown crops, with 352.6 million tons produced in 2017 and more than 100 million hectares of land under cultivation globally, with about half of that in the United States and Brazil [10], these effects have resulted in a considerable drop in yield (24–50%) at different sites and times in greenhouse and field studies [10]. Due to a lack of water, nutrient availability, and nutrient uptake, drought-stressed soybeans are often shorter with smaller leaves. As a result, the production of soybeans is seriously threatened by rising global temperatures and shifting precipitation patterns, especially in places that receive little or no irrigation [11,12]. It is well known that the yield of soybeans can decrease by more than 50% during dry or droughty circumstances, resulting in significant financial losses for farmers and producers [13]. Therefore, drought is a serious climatic risk that necessitates adequate mitigation measures in order to maintain the global soybean supply.

Plant scientists must assess various approaches to enhancing plant growth and development using biochemicals, plant-growth-promoting microorganisms, proteomics, genomics, and breeding techniques. Among these, PGPR and biochemicals such as melatonin are considered more efficient and eco-friendly techniques to improve plant growth and development under stressful conditions. Plant-growth-promoting bacteria can be found in the rhizosphere in the form of colonies around the plant roots (rhizosphere) and play a vital role in sustainable agriculture [14] through direct or indirect methods, which are mainly expressed by directly promoting the absorption of nutrients through the modulation of the levels of plant hormones. The most important and studied direct mechanisms are nitrogen fixation such as *Enterobacter cloacae* [15], *Pseudommonas*, *Bacillus*, *Burkholderia*, and *Pantoea* [16], and the solubilization of inorganic phosphate by *Acinetobacter calcoaceticus* [17], *Bacillus mageterium*, and *Bacillus polymyxa* [18]. Moreover, many of these microbes are reported to have significant production of phytohormones such as auxins, produced via *Acienetobacter ursingii* and *Buttiauxella noackiae* [19]; ABA-producing *Bradyrhizobium japonicum* [20]; GA-producing bacteria such as *Bacillus cereus* and *Bacillus albus* [21]; and enzyme 1-aminocyclopropane-1-carboxylate (ACC) deaminase producing *Achromobacter* sp [22] and *Rhizobium leguminosarum* [23]. The use of PGPR has been shown to be an environmentally responsible way to promote plant development and agricultural yields [14,24,25]. Inducing resistance against plant infections, controlling hormonal and nutritional balance, and solubilizing minerals for simple uptake by plants are some of the processes of PGPR. Additionally, PGPR exhibits synergistic and antagonistic interactions with microorganisms in the bulk soil and the rhizosphere, which indirectly accelerate plant growth rates [26,27,28,29]. Similar to melatonin, which is found in all living things, including bacteria and humans, melatonin (MET, N-acetyl-5-methoxytryptamine) is a widely distributed and highly conserved chemical that is crucial to numerous developmental processes [30]. Furthermore, MET has been reported to improve plant tolerance to environmental stresses such as salinity, drought, heavy metals, and pathogen infection [31,32,33]. Exogenous melatonin administration to plants reduces stress-responsive gene expression and boosts antioxidant enzyme activity [2]. Under various abiotic stresses, overproduced reactive oxygen and nitrogen species were detoxified, leading to stress tolerance and eventually increased plant growth [34,35]. Therefore, the current study aimed to evaluate the synergistic association of PGPR and melatonin to validate their potential role either individually or in co-inoculation on soybean plants to mitigate drought stress through physiological, biochemical, and molecular signaling. This evaluation may reveal a vital role of melatonin and rhizobacteria in mitigating drought stress in soybeans through an eco-friendly and low-cost biofertilizer that improves abiotic-stress tolerance.

## 2. Results

### 2.1. Isolation, Screening, and Identification

Ten isolates were randomly selected for multi-PGP trait screening from previously isolated isolates by Khan et al. [36]. The isolates were analyzed for their capability to exhibit different PGP traits such as production of EPS, siderophore, phosphate, IAA, and resistance to PEG. Among these isolates, PLT16 displayed a more dominant growth pattern in LB, EPS, and siderophore production (Figure S1A–C). The Salkowski test for IAA production showed a higher production of IAA, whereas the three other isolates, ARE1, 222-5, and MS02, demonstrated slight IAA capability (Figure S1D). Based on these observations, the isolate PLT16 was further screened at different concentrations of PEG stress (0%, 3%, 6%, 9%, and 12%) in Luria–Bertani (LB) broth. The PLT16 showed resistance up to 9% of PEG stress (Figure S2), and the further increase in the PEG concentration and time period decreased its resistance capability. In addition, the 16S rRNA sequence of PLT16 was identified as *Lysinibacillus fusiformis* and showed closer identity to *Lysinibacillus* sp. and *Lysinibacillus sphearicus.* The nucleotide sequence of PLT16 was submitted to the NCBI GeneBank Database under accession number OP328329 (Figure S3).

### 2.2. In Vitro IAA and Organic-Acid Production under PEG Stress

**Figure 1:**
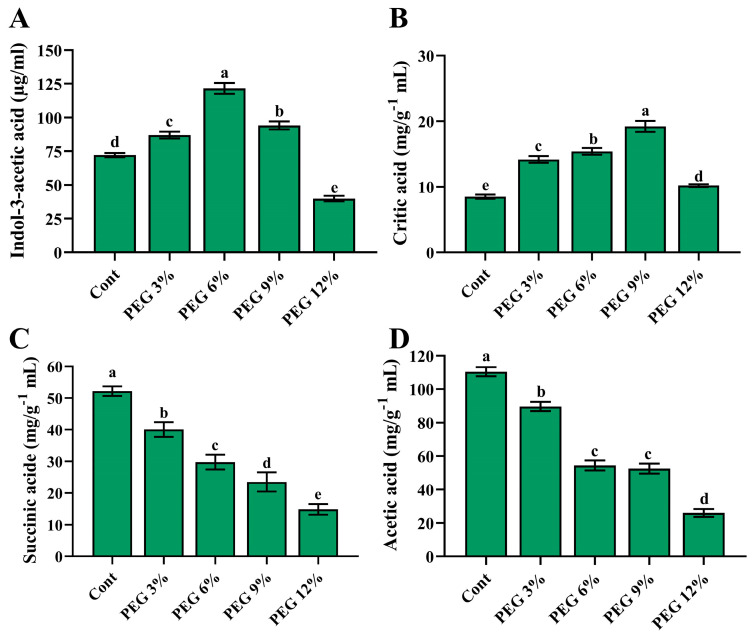
In vitro quantification of IAA and organic acids produced by isolate PLT16. (**A**) GC-MS-SI-M analysis of IAA content in the culture broth, (**B**) critic acid, (**C**) succinic acid, and (**D**) acetic acid through high-performance liquid chromatography (HPLC). Each data point is the mean of three replicates. The standard error of the mean is represented as error bars. Different letters on each bar represent significant differences as evaluated by DMRT and *t*-test at *p* ≤ 0.05.

The culture filtrate of isolate PLT16 was quantified for IAA and organic-acid production using GC/MS. The results show that isolate PLT16 was capable of producing a higher level of IAA under 6% PEG, followed by 9% and 3% PEG, whereas with 12% PEG, the level of IAA production was reduced by 28.4% compared with the control (Figure 1A). Moreover, the production of three organic acids (critic acid, succinic acid, and acetic acid) was detected by PLT16 under PEG. A higher critic-acid level was found under 9% PEG, followed by 6% and 3%, whereas under 12% PEG, its level was reduced by 26.5% compared to PEG 9% (Figure 1B). However, succinic acid and acetic acid showed significantly higher levels in the control, where their production levels decreased with increasing concentrations of PEG (Figure 1C,D).

### 2.3. Isolate PLT16 and Melatonin to Regulate Soybean Growth under Drought Stress

**Figure 2:**
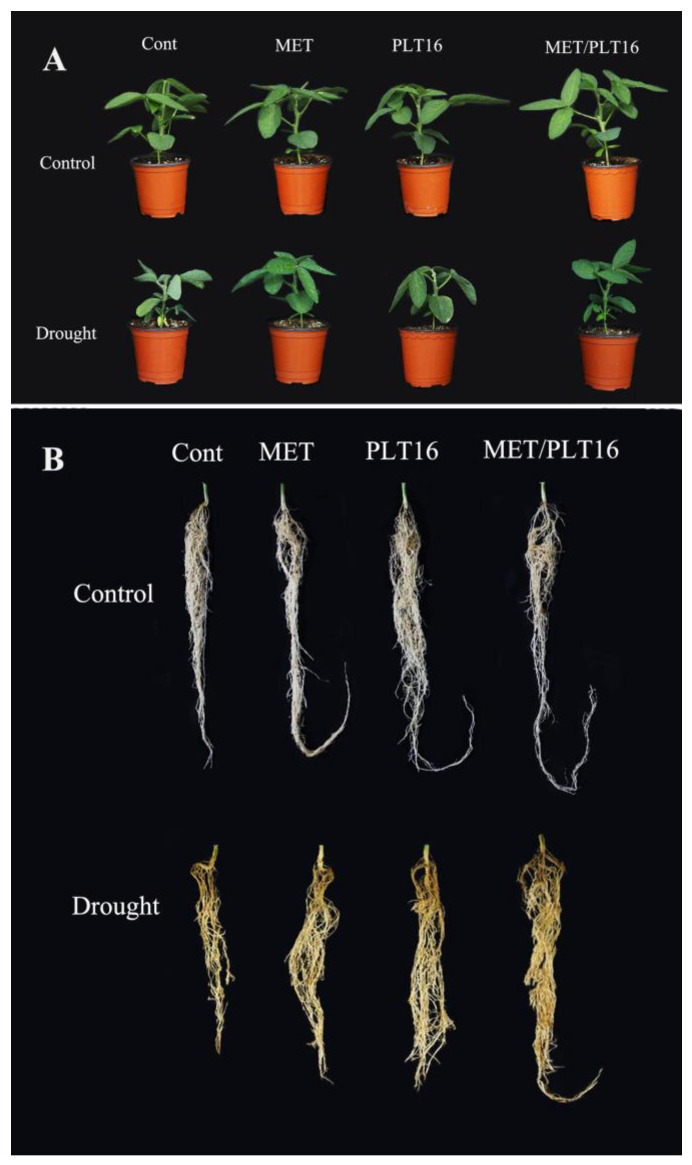
Effects of isolate PLT16 and melatonin application on phenotypical visualization of (**A**) the shoot and (**B**) the root of soybean plants under drought stress.

To validate the synergistic effects of isolate PLT16 and MET on soybean-plant growth and development, growth attributes such as biomass, root/shoot length, and chlorophyll content were measured. The results show that under normal conditions, a moderate increase was found in MET- and PLT16-inoculated plants compared to the control plants, whereas the combined inoculation of MET/PLT16 significantly increased plant growth and biomass compared to the control plants. However, under drought stress, a significant reduction occurred in soybean plants’ shoot/root length, plant biomass, and chlorophyll content compared to MET- or PLT16-treated plants, whereas plants treated with the combined inoculation of MET/PLT16 significantly increased soybean plants’ FW (38.5%), DW (42.7%), shoot length (33.1%), root length (28.6%), and total chlorophyll content (26.3%), followed by individual MET and PLT16 inoculations, when compared to the control plants (only drought) (Figure 2; Table 1). Furthermore, drought stress inhibited chlorophyll pigments, and a decrease was observed in chl a (44.6%), chl b (27.9%), and carotenoids (17.5%) compared with the control (only water-treated plants). However, an increase in the chlorophyll parameters chl a (12.8%), chl b (11.5%), and carotenoids (16.5%) was observed by co-inoculation of MET/PLT16 under drought stress (Figure S4).

### 2.4. Isolate PLT16 and Melatonin Scavenge Reactive Oxygen Species (ROS) and Activate Antioxidant Enzymes

**Figure 3:**
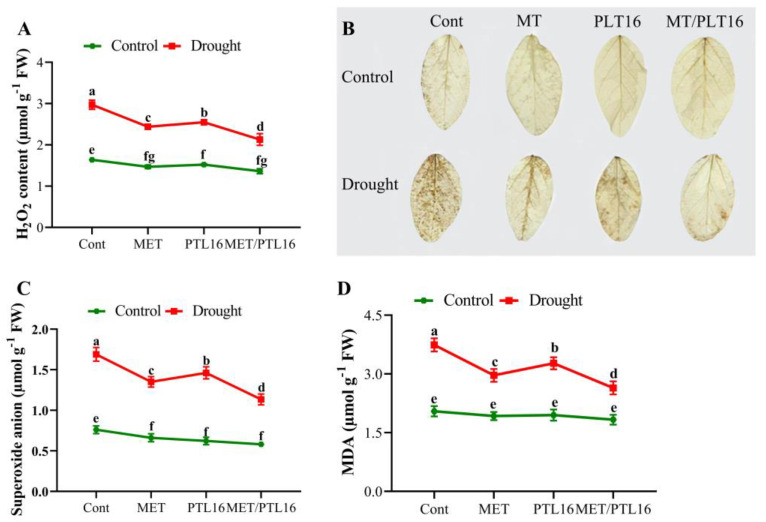
Effects of melatonin and isolate PLT16 application on (**A**) hydrogen peroxide, (**B**) DAB staining, (**C**) superoxide anion, and (**D**) MDA levels in soybean plants under drought stress. Each data point is the mean of three replicates. Error bars represent the standard error of the mean. Bars with different letters are significantly different from each other as per Duncan’s multiple range test at *p* ≤ 0.05.

Under stressful conditions, plants produce ROS such as O^2−^ and H_2_O_2_ as stress signals, but their over-accumulation can cause damage to plant cells. Therefore, the current results show that there was a significant increase in H_2_O_2_ (48.4%) and O^2−^ (61.3%) compared to the control plants. However, the co-inoculation of MET/PLT16 significantly reduced these over-accumulations in H_2_O_2_ and O^2−^ by 34.1% and 29.6%, respectively, followed by individual MET and PLT16 inoculation, when compared to the control plants (only drought) (Figure 3A–C). Moreover, a significant reduction in MDA level by 41.2% in the MET/PLT16 co-inoculated plants was observed under drought stress, followed by the sole MET- and PLT16-treated plants, where the drought stress caused a significant Increase in MDA level by 48.1% compared to the control plants (well-watered) (Figure 3D).

**Figure 4:**
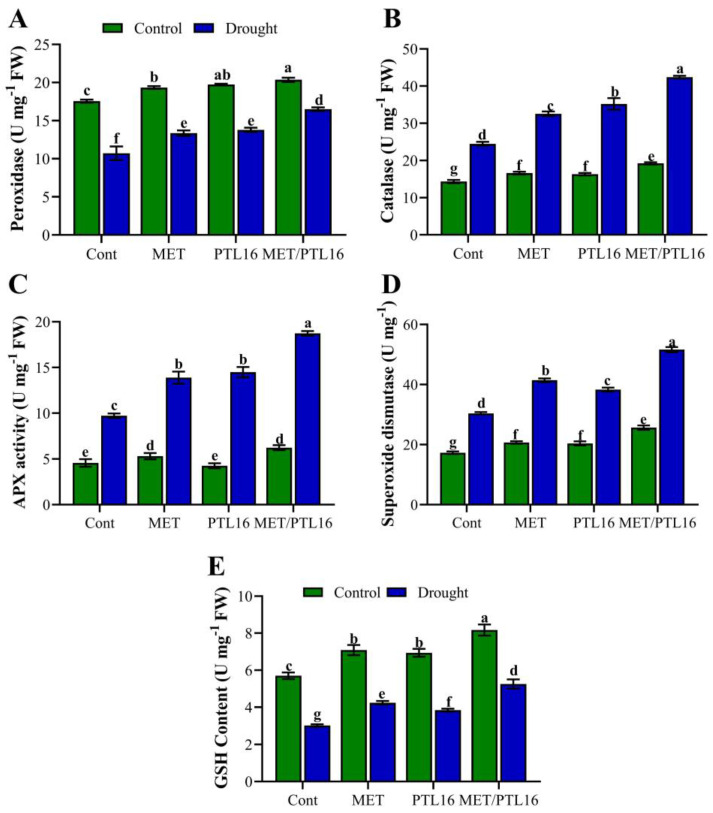
Effects of isolate PLT16 and melatonin application on antioxidant activities: (**A**) peroxidase, (**B**) catalase, (**C**) APX, (**D**) superoxide dismutase, and (**E**) GSH activity in soybean plants under drought stress. Each data point is the mean of three replicates. Error bars represent the standard error of the mean. Bars with different letters are significantly different from each other as per Duncan’s multiple range test at *p* ≤ 0.05.

In response to the overproduction of ROS, plants try to maintain redox homeostasis and protect from oxidative damage by activating antioxidant enzymes. The present study also investigated the effects of melatonin and PLT16 on antioxidant activity under drought stress. The results show that drought stress significantly reduced the POD content by 33.2% compared to the control plants (well-watered), whereas the plants co-inoculated with MET/PLT16 significantly improved this reduction in POD by 23.7% compared to the control plants (only drought) (Figure 4A). Furthermore, an increase in CAT, APX, and SOD was found in the drought-treated plants compared to the control plants (well-watered), whereas co-inoculation of MET and PLT16 caused a further increase in CAT (52.4%), APX (36.5%), and SOD (38.7%), followed by individual MET and PLT16 inoculations, compared to the control plants (only drought) (Figure 4B–D). Furthermore, the non-enzymatic antioxidant GHS was significantly increased by 13.4% in MET/PLT16-treated plants under normal conditions compared to the control plants. However, the drought stress caused a significant decrease in GSH content by 46.3% compared to the control plants (well-watered), whereas the combined application of MET and PLT16 improved this reduction in the non-enzymatic antioxidant (GSH) by 27.1% compared to the control plants (only drought), followed by sole MET and PLT16 application (Figure 4E).

### 2.5. Effects of Isolate PLT16 and Melatonin on Water Status (RWC) and Proline Content under Drought Stress

**Figure 5:**
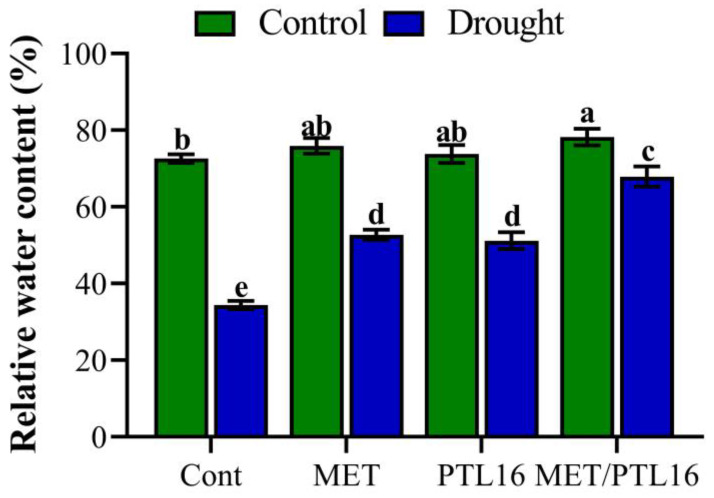
Effects of isolate PLT16 and melatonin application on the relative water content of soybean plants under drought stress. Each data point is the mean of three replicates. Error bars represent the standard error of the mean. Bars with different letters are significantly different from each other as per Duncan’s multiple range test at *p* ≤ 0.05.

Relative water content is the key determinant of water status in plants. The results indicate a non-significant difference in leaf RWC under normal conditions (no stress), whereas drought stress significantly reduced the leaf RWC by 49.7% compared to the control plants (well-watered). However, plants co-inoculated with MET and PLT16 significantly improved the leaf water status by 33.2% compared to the control plants (only drought), followed by individual inoculations of MET and PLT16 (Figure 5). Moreover, the proline content showed a significant increase of 52.6% in drought-treated plants compared to the control plants (well-watered), whereas co-inoculation of MET and PLT16 caused a reduction in the proline content by 38.9% compared to the control plants (only drought), followed by their individual inoculations (Figure S5).

### 2.6. Effect of Isolate PLT16 and Melatonin on Phytohormones ABA, JA, and SA

**Figure 6:**
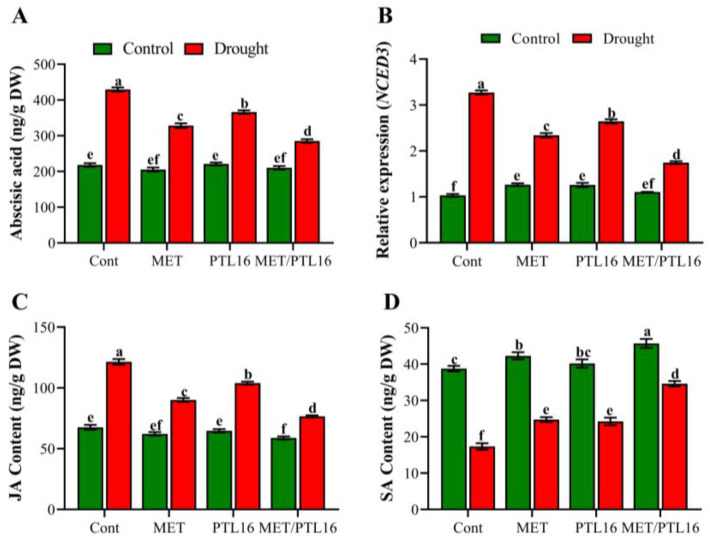
Effects of isolate PLT16 and melatonin application on (**A**) abscisic acid (ABA) content, (**B**) relative expression of the ABA biosynthesis gene *gmNCED3*, (**C**) endo-jasmonic-acid (JA) content, and (**D**) endo-salicylic acid (SA) in soybean plants under drought stress. Each data point is the mean of three replicates. Error bars represent the standard error of the mean. Bars with different letters are significantly different from each other as per Duncan’s multiple range test at *p* ≤ 0.05.

Abscisic acid, a stress-signaling hormone, regulates osmotic-stress tolerance. Our results indicate that under drought stress, the ABA content significantly increased by 58.4% compared to the control plants (well-watered), whereas co-inoculation of MET and PLT16 significantly reduced the ABA level by 37.4% compared to the control plants (only drought) (Figure 6A). Moreover, this was confirmed by the downregulation of the ABA biosynthesis gene *NCED3* (by 27.9%) in plants co-inoculated with MET and PLT16 compared to the control (only drought), followed by their individual MET and PLT16 inoculations (Figure 6B). A similar pattern of increase was observed in the JA level. Under drought stress, a higher level of JA (67.5%) was found compared to control plants (well-watered), whereas the MET and PLT16 inoculations reduced this increase in JA accumulation by 38.6% compared to the control plants (only drought) (Figure 6C). Moreover, a slight increase in SA was found under co-inoculation of MET and PLT16, whereas a non-significant difference was observed in individual MET, PLT16, and control plants (well-watered). However, the SA content was significantly reduced by 62.5% in drought-treated plants compared to control plants (well-watered), whereas the plants co-inoculated with MET and PLT16 demonstrated a significant increase in SA level by 32.1% compared to the control plants (only drought), followed by their individual inoculations with MET- and PLT16-treated plants (Figure 6D).

### 2.7. Effects of Isolate PLT16 and Melatonin on Endo-Melatonin Regulation under Drought Stress

**Figure 7:**
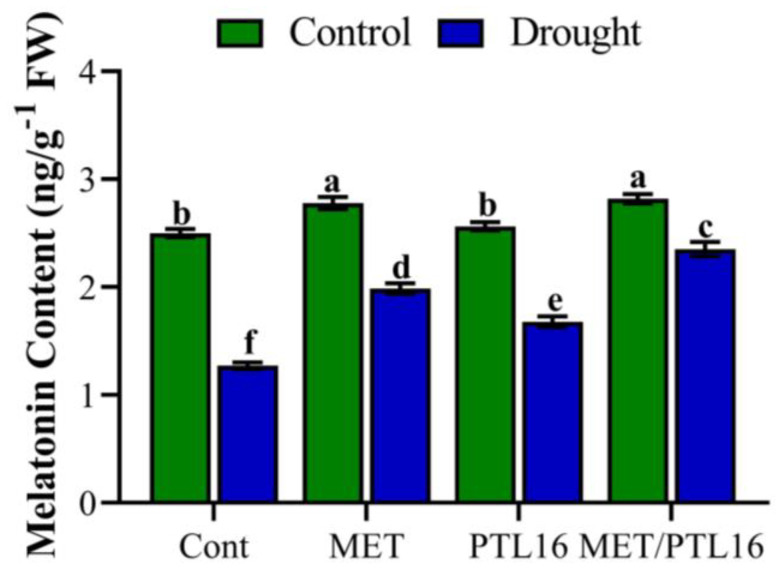
Effects of isolate PLT16 and melatonin application on endogenous melatonin content in soybean plants under drought stress. Each data point is the mean of three replicates. Error bars represent the standard error of the mean. Bars with different letters are significantly different from each other as per Duncan’s multiple range test at *p* ≤ 0.05.

In the present study, we also evaluated the effects of isolate PLT16 and melatonin on endogenous melatonin content under drought stress. The result shows that under normal conditions, a non-significant difference was found in endo-MET content in the control and PLT16-treated plants, whereas the co-inoculation of MET and PLT16 significantly increased the endo-MET content by 9.8% compared to the control and PLT16-treated plants, and no difference was observed in the MET-treated plants. Moreover, drought-stress-treated plants caused a significant reduction in endo-MET content by 34.8% compared to the control plants (well-watered), whereas co-inoculation of MET and PLT16 improved this reduction in endo-MET content by 22.3% compared to the control plants (only drought), followed by individual inoculation of MET and PLT16 (Figure 7).

### 2.8. Effects of PLT16 and Melatonin on Plant Organic Acid and Micronutrient Content under Drought Stress

**Figure 8:**
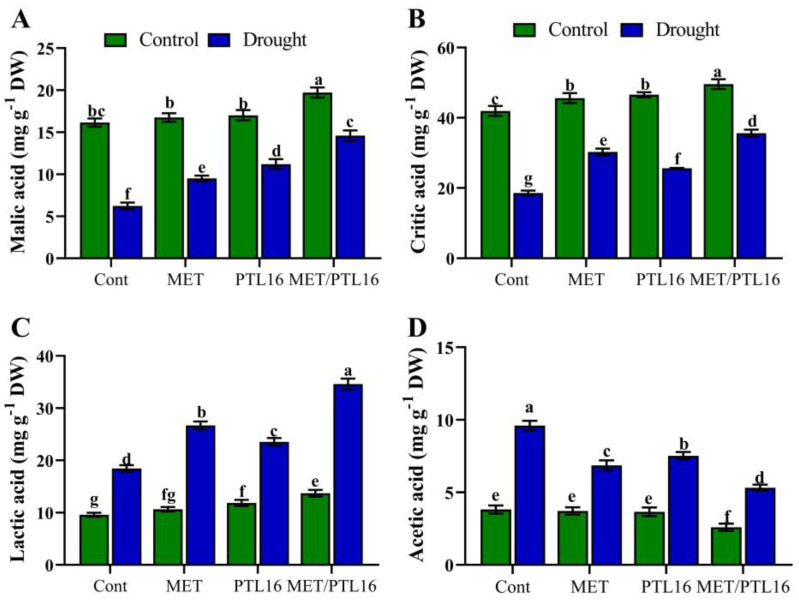
Effects of isolate PLT16 and melatonin application on organic-acid content in (**A**) malic acid, (**B**) critic acid, (**C**) lactic acid, and (**D**) acetic acid in soybean plants under drought stress. Each data point is the mean of three replicates. Error bars represent the standard error of the mean. Bars with different letters are significantly different from each other as per Duncan’s multiple range test at *p* ≤ 0.05.

Organic acid plays a vital role in various cellular processes, such as balancing redox homeostasis, improving stomata function, and supporting the ion gradient. The results demonstrate that under normal conditions (no stress), malic acid and critic acid were significantly increased by co-inoculation of MET and PLT16, whereas drought stress caused a reduction in malic acid (71.3%) and critic acid (48.5%) compared to the control plants (well-watered). The co-inoculation of MET and PLT16 improved malic acid and critic acid by 38.1% and 26.9%, respectively, compared to the control plants (only drought), followed by individual inoculation of MET and PLT16 (Figure 8A,B). Moreover, lactic acid was increased by 19.6% in drought-treated plants, whereas the co-inoculation of MET and PLT16 further enhanced the lactic acid content by 36.4% compared to the control (only drought) plants. Additionally, drought stress significantly increased the acetic-acid content by 51.2% compared to the control plants (well-watered), whereas co-inoculation of MET and PLT16 reduced the acetic-acid content by 28.6% compared to the control plants (only drought), followed by the individual inoculations of MET and PLT16 (Figure 8C,D).

**Figure 9:**
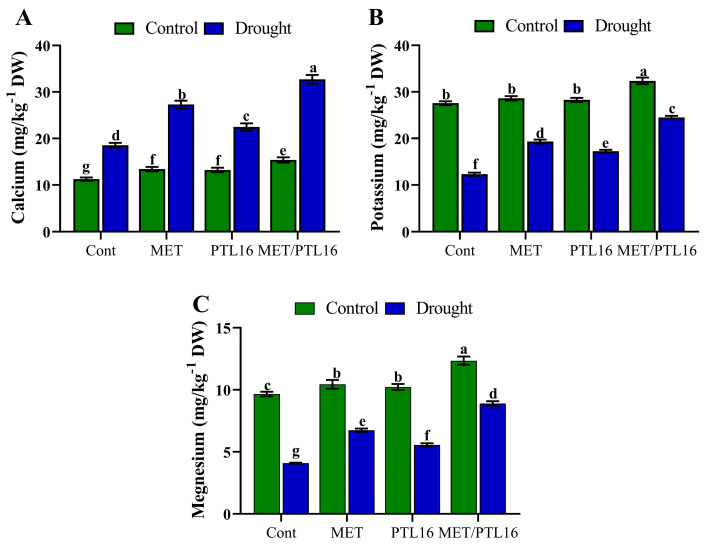
Effects of isolate PLT16 and melatonin application on content of nutrients such as (**A**) calcium, (**B**) potassium, and (**C**) magnesium in soybean plants under drought stress. Each data point is the mean of three replicates. Error bars represent the standard error of the mean. Bars with different letters are significantly different from each other as per Duncan’s multiple range test at *p* ≤ 0.05.

Inductively coupled mass spectrometry (ICP) for the analysis of calcium, potassium, and magnesium was performed. The results suggest that under normal conditions (no stress), the co-inoculation of MET and PLT16 significantly increased calcium, potassium, and magnesium compared to the control plants. However, under drought stress, the calcium content was slightly increased by 9.6% compared to the control plants, whereas the plants treated with MET and PLT16 caused a further increase in calcium content of 29.8% compared to the control plants (only drought). Moreover, a significant decrease in potassium (47.6%) and magnesium (28.1%) in drought stress-treated plants was found compared to control plants (no stress), whereas the co-inoculation of MET and PLT16 application enhanced this decrease in calcium, potassium, and magnesium content by 26.4% and 21.5%, respectively, compared to the control plants (only drought) (Figure 9A–C).

### 2.9. Effects of Isolate PLT16 and Melatonin on Transcriptional Regulation under Drought Stress

**Figure 10:**
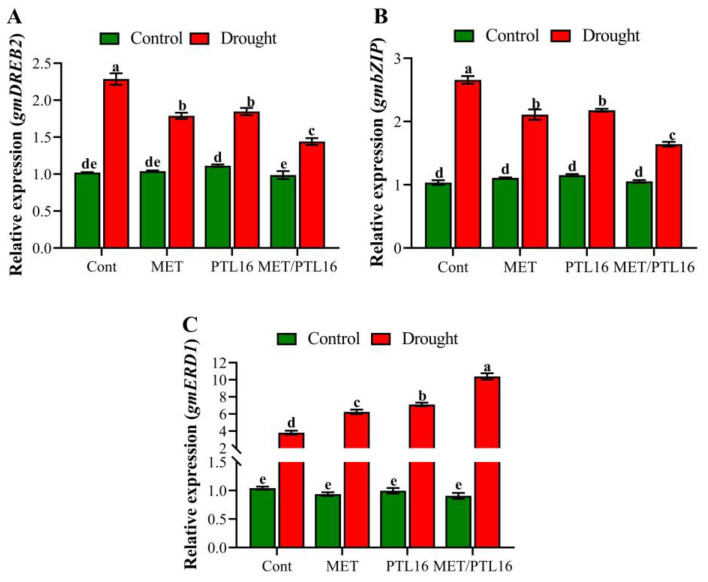
Effects of melatonin and isolate PLT16 application on stress-response transcription factors (**A**) DREB2, (**B**) bZIP, and (**C**) ERD1 in soybean plants under drought stress. Each data point is the mean of three replicates. Error bars represent the standard error of the mean. Bars with different letters are significantly different from each other as per Duncan’s multiple range test at *p* ≤ 0.05.

The alleviating effects of PLT16 and MET under drought stress were determined by investigation of drought-related expression of genes such as *GmDREB2*, *GmERD1*, and *GmbZIP* in soybean plants. The results show that in the absence of stress, a non-significant up-regulation was found in the relative expression of *GmDREB2*, *GmERD1*, and *GmbZIP* under co-inoculation and individual inoculations of PLT16 and MET compared to the control plants. However, under the drought-stress condition, the relative expression of *GmDREB2* and *GmbZIP* was significantly increased by 97.6% and 112.5%, respectively, compared to the control plants, whereas the co-inoculation of MET and PLT16 significantly reduced this up-regulation in *GmDREB2* and *GmbZIP* by 53.4% and 67.1%, respectively, compared to the control plants (drought-treated), followed by the individual MET and PLT16 treatments (Figure 10A,B). Furthermore, under drought stress, a significant up-regulation was found in the relative expression of *GmERD1* compared to the control plants, whereas the combined application of MET and PLT16 further enhanced the up-regulation of *GmERD1* by 131.5% compared to the control plants (drought-treated), followed by their individual inoculations (Figure 10C).

## 3. Discussion

In recent years, exogenous substances have been widely used to improve plant stress resistance and crop yield through breeding, genomics, proteomics, and biochemicals. Among these, exogenous melatonin and PGPR have emerged as research interests in plant science and to alleviate the effects of abiotic stress [37,38]. In the present study, we investigated the potential role of PGPR along with melatonin in soybean plants under drought stress, and we successfully isolated many PGPR characteristics in our laboratory. Moreover, on comparing the growth patterns of various isolates, PLT16 demonstrated the most impressive EPS, phosphate-solubilizing, and siderophore-production properties. PGPR promotes plant growth by colonizing plant roots and helps plants grow in a number of ways, such as by producing siderophore for the availability of iron uptake; by phosphate solubilization, which makes it easier for plants to take up phosphorus; by producing exopolysaccharide, which is responsible for making biofilm; by fixing nitrogen; by making 1-aminocyclopropane-1-carboxylate deaminase; and by making phytohormones [37].

Abiotic stressors, such as drought, heat, ultraviolet rays, and cold, significantly impede plant growth through a variety of processes and reduce crop output. Among these, drought is complicated, and its periodic occurrence negatively affects agriculture, the economy, water supplies, and ecosystems [3]. Additionally, it causes alterations in the colloidal–chemical composition of the cytoplasm, resulting in a lack of water, protein breakdown, and a reduction in the number of organic compounds collected in plants, which leads to osmotic stress induced by a decrease in plant growth and development, biomass, and chlorophyll pigments [4,5]. Therefore, the present study shows that co-inoculation of PLT16 and MET significantly improved the soybean plants’ growth and development, biomass, and chlorophyll content, and reduced the level of ROS to mitigate drought stress. Similarly, Khalilpour et al. reported an improvement in pistachio-plant growth and development by PGPR under drought stress [39], and melatonin mitigated drought stress in in tall fescue grass [2]. Plants produce ROS in response to stress, and higher concentrations can lead to oxidative stress. Furthermore, oxidative stress is the most detrimental environmental stress for plants. Melatonin and PGPR operate as free-radical scavengers, directly scavenging ROS and boosting mitochondrial oxidative phosphorylation efficiency [37]. The possibility of oxidative damage and an increase in MDA levels due to an increase in ROS generation is widely recognized [40]. Plants respond to ROS by energizing antioxidant enzymes such as SOD, POD, CAT, and GSH. In order to maintain redox homeostasis under drought stress, co-inoculation of melatonin and PLT16 significantly improves antioxidant capabilities to scavenge ROS and enhances relative water and proline content. Similar outcomes were suggested in soybean plants and in pistachio plants [14,39].

Furthermore, excessive levels of ABA accumulation can promote the production of ROS and result in oxidative damage [41]. The MET and PLT16 administrations reduced ABA accumulation under stressful circumstances. This was demonstrated by the decreased expression of *NCED3* and the perhaps increased expression of genes associated with ABA catabolism, *CYP707A1* and *CYP707A2*, in soybean plants under drought stress. Similarly, [42,43] reported an increase in ABA content during drought stress. Additionally, increased endogenous JA buildup together with decreased biomass and photosynthesis were confirmed in soybean and rice grown under drought and metal stress [44,45]. Similar conclusions were reached by Li et al. [40] and Arnao et al. [34]. They found that melatonin decreased the amount of ABA by controlling the genes for ABA breakdown and production. Furthermore, salicylic acid is essential for the defensive signals that plants produce in response to abiotic stress. Although SA and melatonin have not been directly compared, they share the same metabolic pathway and precursor (chorismic acid) [34]. They have a significant impact on how biotic and abiotic stresses affect plants physiologically and molecularly. Recent studies have revealed that some plants retain melatonin as a protective measure against abiotic challenges, including salt and drought [46,47].

The drought tolerance of plant species is affected by a change in the content of different organic compounds because of environmental-stress conditions. Organic acids are involved in several biological processes, are structural constituents of the cell, and act as a metabolic resource [48]. In our research, we assessed the effect of co-inoculation of bacteria PLT-16 and melatonin on the metabolism of organic-acid content and micronutrients under drought stress. The results indicate that the co-inoculation of PLT-16 and exogenous melatonin was affected similarly to [49] organic content by an increase in both of them compared to the control as a result of drought stress. Ref. [50] used some nanoparticles, which are micronutrients, in soybean under drought stress and found that micronutrients were able to reduce drought effects and help uptake micronutrients. Moreover, root exudates are mostly made up of organic acids (OAs), which are intermediates in the tricarboxylic-acid (TCA) cycle of cellular metabolism. Several environmental stresses cause plants to produce OAs and release them through their roots [51]. Moreover, both melatonin and PGPB, which stimulate plant growth, have great potential for ameliorating the negative outcomes of nutrient deprivation. Our current study found a significant rise in calcium, potassium, and magnesium levels on co-application of MET and PLT16 under both control and drought stress compared to after applying either factor alone. Plants with higher calcium concentrations are better able to detoxify environmental stress, resulting in increased antioxidant activity [52]. Moreover, potassium influences osmotic control, stomatal movement, and the transfer of photosynthetic products [53,54]. In contrast, melatonin prevented chlorophyll loss by elevating magnesium levels. As a result, oxidative stress was prevented and thylakoid membranes were protected [53].

Numerous genes susceptible to drought contain a dehydration-responsive element (*DREB*). Transcription factors (TFs) from the AP2/ERF (Apetala2 and ethylene-responsive factors) family include *DREB2A* and *DREB2B* [55]. Moreover, dehydration significantly upregulated the expression of DREB2A, demonstrating that plants respond to drought stress through a mechanism independent of ABA [56]. Drought activates the dehydration-responsive gene *ERD1*, whereas ABA does not [57]. Plant *bZIPs* have a highly conserved region composed of a leucine zipper and the basic amino acids that surround it. *GmbZIP2* overexpression in soybean hairy root systems and Arabidopsis boosted plant resilience to abiotic stresses such as dryness and salt [58]. Melatonin increases the expression of genes associated with the antioxidant system, increasing the tolerance of the plant to the impacts of stress [59,60]. Furthermore, to investigate the response of soybean plants to drought, the expression of various genes, such as *GmDREB2*, *GmERD1*, and *GmbZIP*, was examined in our study. In comparison to control plants, relative expression of *GmDREB2* and *GmbZIP* was significantly upregulated by 97.6% and 112.5%, respectively, under water-limitation conditions; however, co-inoculation with MET and PLT16 significantly reduced this up-regulation by 53.4% and 67.1%, respectively (drought-treated) (Figure 10A,B). However, the combined application of MET and PLT16 increased *GmERD1* expression by 131.5% compared to the control plants (drought-treated), followed by individual MET and PLT16 inoculations (Figure 10C). In conclusion, the application of exogenous melatonin in conjunction with the co-inoculation of PGPB can be an efficient protectant that increases drought tolerance in soybean plants by increasing antioxidant enzymes and decreasing oxidative damage.

## 4. Materials and Methods

### 4.1. Isolation, Screening, and Identification

In the current study, various rhizospheric bacterial species, previously isolated by Khan et al. from Pohang, Republic of Korea (35°55′40.3″ N 129°31′00.5″ E), were used [36]. The isolates were screened for different plant-growth-promoting traits, including the ability to produce exopolysaccharide and indole-3-acetic acid, phosphate solubilization, and resistance to PEG stress. For all isolates, the Salkowski reagent was used for the production of IAA following the method of [61], and the Congo-red assay was used for the formation of EPS [62]. Siderophore production and phosphate solubilization were performed by the method described previously by Schwyn and Neilands [63]. The determination of resistance to PEG stress was performed using five different concentrations of PEG 6000 (0%, 3%, 6%, 9%, and 12%) prepared in LB broth and sterilized at 121 °C for 15 min. Later, 1 mL of culture aliquots was inoculated into 10 mL of sterilized LB broth and incubated at 28 °C. The optimum density of the isolate in each concentration of PEG along with the control was measured every 8 h (8 h, 16 h, and 24 h) using a spectrophotometer at 600 nm.

The PTL16 isolate was chosen for additional research based on the optimal efficiency and survival curve under PEG, EPS, siderophore, phosphate solubilizing, and IAA formation. The isolate was identified using a 16S rRNA-specific primer and genomic DNA, and the amplification was performed using the detailed method reported by Khan and Halo et al. [64]. The homology of the various nucleotide sequences of PLT16 was compared using the NCBI BLAST (on 16 December 2022, https://blast.ncbi.nlm.nih.gov/Blast.cgi)) and EzTaxon (on 16 December 2022, https://www.ezbiocloud.net/) programs, and MEGAx software was utilized for the polygenetic analysis.

### 4.2. Determination of In Vitro Phytohormone and Organic-Acid Production

The IAA capability in the culture filtrate of isolate PLT16 was determined using GC-MS/SIM (5973 Network Mass Selective Detector and 6890N Network Gas Chromatograph; Agilent Technologies, Palo Alto, CA, USA) [36]. Furthermore, for the determination of the organic-acid content in isolate PLT16, the culture broth was filtered through a 0.45 μm Millipore filter (DISMIC-25CS, ADVANTE, Tokyo, Japan), and 10 μL of each sample were injected into a high-performance liquid-chromatography (HPLC) column (waters 600 E; column: RSpakKC-811 (8.0 × 300 mm^2^); eluent: 0.1% phosphoric acid/water; flow rate: 1.0 mL/min; temperature: 40 °C). The retention times and peak areas in the chromatograms were compared with standards from Sigma-Aldrich, St. Louis, MO, USA [65].

### 4.3. Plant-Growth Conditions

The Soybean Genetic Resource Center (Kyungpook National University, Daegu, Republic of Korea) provided soybean (*Glycine max* L.) seeds. The seeds were first surface-sterilized for 5 min with a 2.5% sodium-hypochlorite solution, followed by three washes with autoclaved double-distilled water before sowing seeds in plastic trays with horticultural substrate [66]. The seedlings were cultivated in a growth chamber at an optimum temperature of 24–27 °C, relative humidity of 55–65%, 1000 μEm^2^/s, and a day/night cycle at 14–10 h’. Similar-sized seedlings were chosen at the VC stage (with unrolled unifoliate leaves) and transferred into plastic pots filled with the same autoclave horticultural soil. One seed was retained in each pot during the experiment, and the experiment was carried out with 10 duplicates of each treatment. The soybean seedlings were divided into two sets of treatments: (A) normal condition without any stress, including (i) control plants, (ii) MET (melatonin) treated, (iii) isolate-PLT16 treated, and (iv) MET/PLT16 treated, whereas the (B) group featured drought stress, including (i) control plants (drought-treated), (ii) MET/drought-treated, (iii) isolate-PLT16/drought-treated, and (iv) MET/PLT16/drought-treated.

Furthermore, three days after transplantation into pots, the plants were subjected to a pre-treatment with 100 μm of melatonin in 50 mL of water per plant as previously reported by [66] and 50 mL of isolate PLT16 for 5 days, and were then exposed to drought stress by withholding water until the soil-moisture content dropped to 25–30% FC. The soil-moisture content was measured using DEMETRA, E.M. System Soil Tester, Tokyo, Japan. For isolate-PLT16 inoculation, the isolate was cultured for 3 days in LB broth at 28 °C in a shaking incubator and then centrifuged at 6000 rpm for 10 min. The obtained pellet was suspended in autoclave-distilled water and inculcated into plants. Following the completion of the experiment, growth characteristics (root and shoot length), biomass (fresh and dry weight), and chlorophyll content were determined using the SPAD-502 Chl meter (Konica Minolta, Japan) and instantly frozen in liquid nitrogen before being transported to −80 °C for further investigation.

### 4.4. Determination of Chlorophyll a and b, Carotenoid, Proline, and RWC (Relative Water Content)

Chlorophyll a and b were determined using the method reported in the literature: A fresh plant sample (0.3 g) was ground with 80% acetone and then vertex and incubated at room temperature for 30 min, followed by centrifugation at 10,000× *g* for 10 min. The obtained supernatant was spectrophotometrically recorded at 470, 645, and 663 nm for the determination of chl a and b and carotenoid, respectively [67]. The determination of RWC and proline content was carried out by following detailed method of [66,68,69].

### 4.5. Determination of H_2_O_2_, MDA, and Superoxide-Anion Content

The amount of H_2_O_2_ in the sample was determined using the technique reported previously in the literature [70]. Briefly, the leaf specimen was crushed, extracted, and centrifuged for 15 min at 12,000× *g* with 5 mL of 0.1% trichloroacetic acid (TCA). The supernatant was collected (1 mL), and the absorbance was spectrophotometrically measured at 390 nm after the addition of 1 mL of 1 M potassium iodide and 0.5 mL of 10 M phosphate buffer (pH 7.0). The amount of H_2_O_2_ present was calculated using the extinction coefficient of 0.28 mM cm^−1^ and expressed as mg^−1^ DW. The level of MDA and superoxide-anion content in leaves was determined through the detailed method described in the literature [36,69,71].

### 4.6. Quantification of Antioxidant Enzymes and Non-Enzymatic Antioxidant-Activitiy Quantification

The catalase (CAT) activity was determined using the approach outlined by Halo et al. [72]. It involved the estimation of the reduction in the amount of H_2_O_2_ absorption at 240 nm. The reaction buffer contained 15 mM hydrogen peroxide and 50 mM potassium-phosphate buffer at a pH of 7.0. Then, 100 μL of the enzyme extract were added to the reaction mixture to initiate the reaction. The H_2_O_2_ level in the reaction mixture was measured after 1 min with an extinction coefficient of 40 mM^−1^ cm^−1^, which indicated CAT enzyme activity. To measure the ascorbate-peroxidase (APX) activity, a 100 mg plant sample was extracted with 1 mL of 50 mM phosphate buffer (pH 7.0) containing 1 mM ascorbic acid and 1 mM EDTA. The homogenates were centrifuged at 4830× *g* (4 °C) for 15 min. The supernatant was mixed with phosphate-buffer solution (pH 7.0), 15 mM ascorbic acid, and 0.3 mM H_2_O_2_, and the reaction mixture was read at 290 nm [73]. Furthermore, we measured the ability of superoxide dismutase (SOD) to prevent the photochemical reduction of nitro blue tetrazolium in the presence of a fluorescent dye, where the SOD activity units were determined as the amount of enzyme required to cause 50% inhibition of the reduction of NBT, as monitored at 560 nm [74]. In addition, peroxidase (POD) quantification was done by adding 0.1 mL of the supernatant to a reaction mixture containing 1 mL of 2% H_2_O_2_, 50 mM phosphate buffer (pH 5.5), and 50 mM guaiacol. The phosphate buffer was used as a negative control in the absence of the enzyme. The POD activity was determined as a unit change per minute by measuring the absorbance at 470 nm for three minutes [75]. Moreover, the non-enzymatic antioxidant-glutathione (GSH) content was quantified following the procedure previously described in the literature [76].

### 4.7. Endogenous Phytohormones ABA, JA, and SA

The ABA quantification and extraction were performed as reported in the literature with minor modifications [77]. Briefly, endo-ABA was extracted from 0.3 g of freeze-dried plant sample, and a chromatograph was run with Me-[2H6]-ABA as an internal standard. The fraction was then methylated with diazomethane, and 1 µL of sample was detected by GC-MS (6890 N network gas chromatography, Agilent Technologies) to determine the ABA content. ThermoQuest software (Manchester, UK) was used to monitor signal ions (*m*/*z* 162 and 190 for Me-ABA; *m*/*z* 166 and 194 for Me-[2H6]-ABA) (Table S1). The JA and SA were quantified using the detailed method previously reported in the literature [78] (Tables S2 and S3).

### 4.8. Quantification of Endogenous Melatonin

Endogenous melatonin was extracted and quantified in soybean-plant leaves in accordance with the manufacturer’s instructions using a melatonin ELISA kit (Enzo Life Sciences, Farmingdale, NY, USA) [79,80].

### 4.9. Determination of Micronutrient Uptake in Plants

The micronutrient content in the shoots of plants inoculated or non-inoculated with isolate PLT16 and melatonin was investigated using inductively coupled plasma mass spectrometry (9ICP-MS; Optime 7900DV, Perkin-Elmer, Waltham, MA, USA) [81].

### 4.10. RNA Extraction and Quantitative Real-Time PCR

The RNA-extraction process was performed as reported in the literature with a few minor modifications [82]. Briefly, liquid nitrogen was used to grind 0.1 g of fresh leaf samples, and the crushed samples were instantly placed in an RNase-free E-tube with an extraction buffer containing Tris-HCl, 0.05 M, pH 7.5; NaCl, 0.25 M; EDTA, 20 mM; PVP, 4% (*w*/*v*); and sodium dodecyl sulfate, 1% (*w*/*v*) [83]. NanoQ was used to gauge the quantity and quality of RNA. Furthermore, complementary DNA (cDNA) synthesis and quantitative real-time polymerase chain reaction (qRT-PCR) were also performed. Briefly, 1 µg of RNA was utilized to create cDNA using the BioFACTTM RT kit (BioFACTTM, Republic of Korea) in accordance with the manufacturer’s instructions. In addition, qRT-PCR (EcoTM IlluminaTMm, Azure Biosystems, Dublin, CA, USA) was then used to further evaluate the transcript accumulation using the generated cDNA as a template. The complete list of genes and the accompanying primers for each gene are shown in Table S4. Briefly, a 20 µL reaction mixture including a 2× real-time PCR master mix (BioFACTTM, Daejeon, Republic of Korea), 10 µm of each gene-specific primer, and 100 ng of template cDNA was used. The following conditions were run with 40 cycles of a two-step PCR: denaturation at 95 °C for 15 s, annealing and extension at 60 °C for 30 s, and polymerase activation at 95 °C for 15 min. A “no-template control” was used as the negative control. The expression of each gene was compared with the relative expression of actin as an internal control, and the experiment was conducted in triplicate.

### 4.11. Statistical Analysis

All of the experimental analysis was carried out in triplicate. Duncan’s multiple-comparison method was used to compare mean values. The distributional-range test (DMRT) was performed at a significance level of 0.05 using SAS 9.1. The results were presented graphically using GraphPad Prism version 8.0 (San Diego, CA, USA).

## 5. Conclusions

In the present study, we analyzed the beneficial effects of melatonin and bacteria co-inoculated in soybean under drought stress. We observed that the application of exogenous melatonin and PGPB isolate PLT-16 had a beneficial effect on those under drought stress as a treatment. The co-inoculated treatment of melatonin and bacteria enhanced plant growth, biomass, and chlorophyll content. Furthermore, ROS generation was decreased, antioxidant activity was increased, and the phytohormone ABA was decreased. Organic-acid production and sugar content were also found to have increased. Moreover, nutrient content was improved using our treatment based on co-inoculation of bacteria and exogenous melatonin. Drought-stress mitigation was observed in soybean plants on co-inoculation of PLT-16 and melatonin. The results show that melatonin and microbial inoculation could be used as biofertilizers in drought-stressed areas at the field level. Further, all-genome sequencing of isolate PLT-16 should be conducted to investigate the response of the drought-stress gene at the molecular level.

## Figures and Tables

**Table 1:** Effects of MET/PLT16 application on growth attributes of soybean plants under drought stress.

Treatment	SL (cm)	RL (cm)	FW (g)	DW (g)	Total Chl (mg/g^−1^ DW)
Control (normal condition)					
Cont	18.5 ± 0.21 b	17.8 ± 0.31 c	8.7 ± 0.14 c	5.8 ± 0.13 c	35.2 ± 0.28 c
MET	19.2 ± 0.25 b	20.2 ± 0.27 b	10.2 ± 0.14 b	7.1 ± 0.14 b	39.4 ± 0.41 b
PLT16	19.0 ± 0.21 b	19.5 ± 0.21 b	10.7 ± 0.16 b	7.6 ± 0.14 b	38.7 ± 0.34 b
MET/PLT16	21.5 ± 0.33 a	23.5 ± 0.35 a	12.8 ± 0.21 a	8.9 ± 0.17 a	43.8 ± 0.51 a
Drought					
Cont	11.2 ± 0.15 e	12.6 ± 0.19 e	6.02 ± 0.1 e	3.1 ± 0.12 e	20.2 ± 0.31 f
MET	14.0 ± 0.21 d	15.6 ± 0.24 d	7.3 ± 0.22 d	4.3 ± 0.15 d	28.0 ± 0.0.23 e
PLT16	14.2 ± 0.22 d	15.3 ± 0.25 d	7.1 ± 0.14 d	4.1 ± 0.15 d	26.5 ± 0.28 e
MET/PLT16	16.5 ± 0.24 c	17.0 ± 0.21 c	8.5 ± 0.16 c	5.2 ± 0.15 c	32.4 ± 0.34 d

The treatment includes control (without any treatment or stress), MET (melatonin treated), PLT16 (isolate *Lysinibacillus fusiformis*), and MET/PLT16 (melatonin + isolate), whereas the measurement includes SL (shoot length), RL (root length), FW (fresh weight), DW (dry weight), and total Chl (total chlorophyll content mg/g^−1^ of dry weight). Each data point is the mean of at least three replicates. The mean ± standard error followed by the different letter(s) are significantly different from each other, as evaluated by DMRT.

## Data Availability

All of the data are available in the manuscript.

## Supplementary Materials

The following supporting information can be downloaded at: https://www.mdpi.com/article/10.3390/ijms24108489/s1.
