# Supplementary material for: Synergistic Effect of Melatonin and Lysinibacillus fusiformis L. (PLT16) to Mitigate Drought Stress via Regulation of Hormonal, Antioxidants System, and Physio-Molecular Responses in Soybean Plants
Source: Int J Mol Sci. 2023 May 9;24(10):8489. doi: 10.3390/ijms24108489 (PMC10218646; doi:10.3390/ijms24108489)
Supplement: Supplementary file 1 [file ijms-24-08489-s001.zip › ijms-2342080-supplementary.pdf]

Synergistic Effect of Melatonin and *Lysinibacillus fusiformis* L. (PLT16) to Mitigate Drought Stress via Regulation of Hormonal, Antioxidants System, and Physio-Molecular Response in Soybean Plants

Muhammad Imran [1,2,†], Clems Luzolo Mpovo [2,†], Muhammad Aaqil Khan [3], Shifa Shaffique [2], Daniel Ninson [2], Saqib Bilal [4], Murtaza Khan [5], Eun-Hae Kwon [2], Sang-Mo Kang [2], Byung-Wook Yun [2] and In-Jung Lee [2,*]

[1] Biosafety Division, National Institute of Agriculture Science, Rural Development Administration, Jeonju 54874, Republic of Korea; m.imran02@yahoo.com
[2] Department of Applied Biosciences, Kyungpook National University, Daegu 41566, Republic of Korea
[3] Department of Chemical and Life Sciences, Qurtuba University of Science and Information Technology, Peshawar 24830, Pakistan
[4] Natural & Medical Sciences Research Center, University of Nizwa, Nizwa 616, Oman
[5] Department of Horticulture and Life Science, Yeungnam University, Gyeongsan 38541, Republic of Korea
* Correspondence: ijlee@knu.ac.kr; Tel.: +82-53-950-5708
† These authors contributed equally to this work.

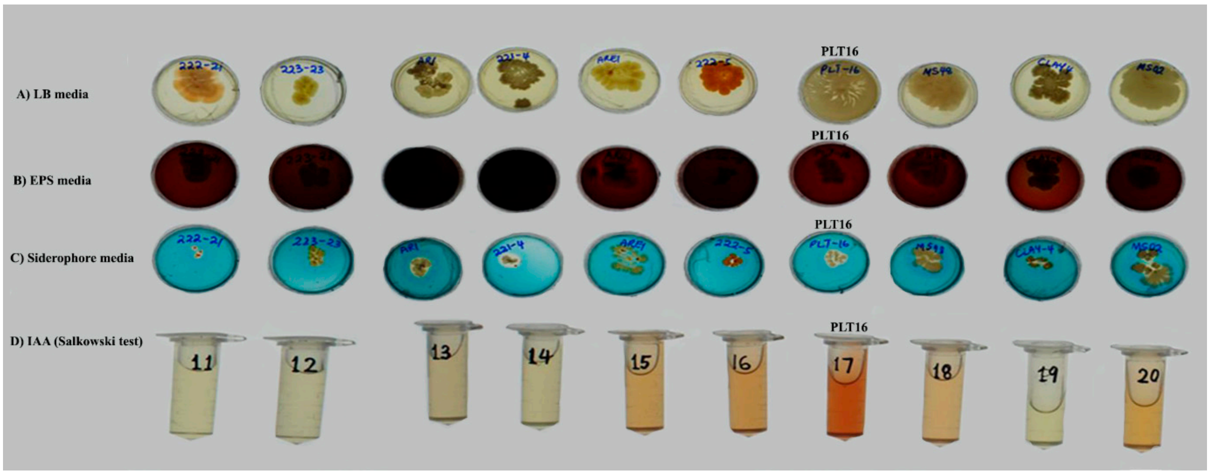

Figure S1. Bacterial isolates were assessed for a beneficial role in PGP activities. (A) LB media plates; (B) exopolysaccharide (EPS) activity on Congo red medium; (C) chromeazurol'S agar plates for siderophore production; (D) Salkowski reagent assay for IAA production.

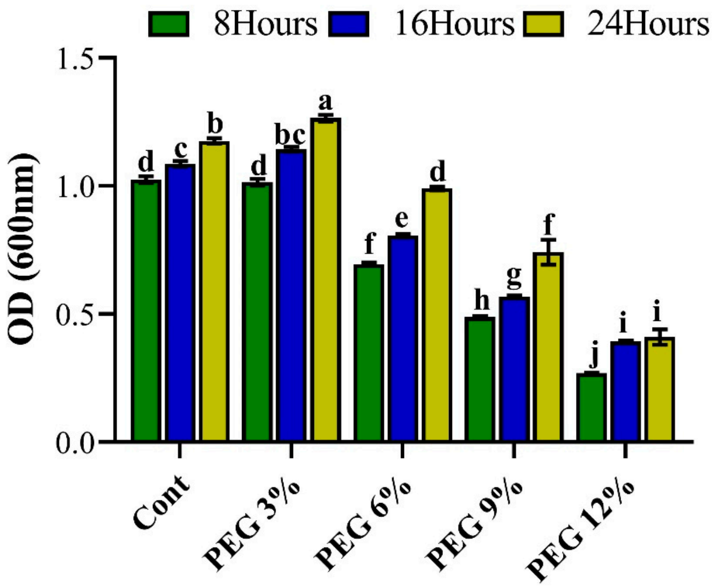

Figure S2. Survival tendency of isolate PLT16 on LB media supplemented with 0, 3, 6, 9 and 12% of PEG for 42h. The growth was examined using spectrophotometer at 600 nm. Each data point is the mean of three replications.

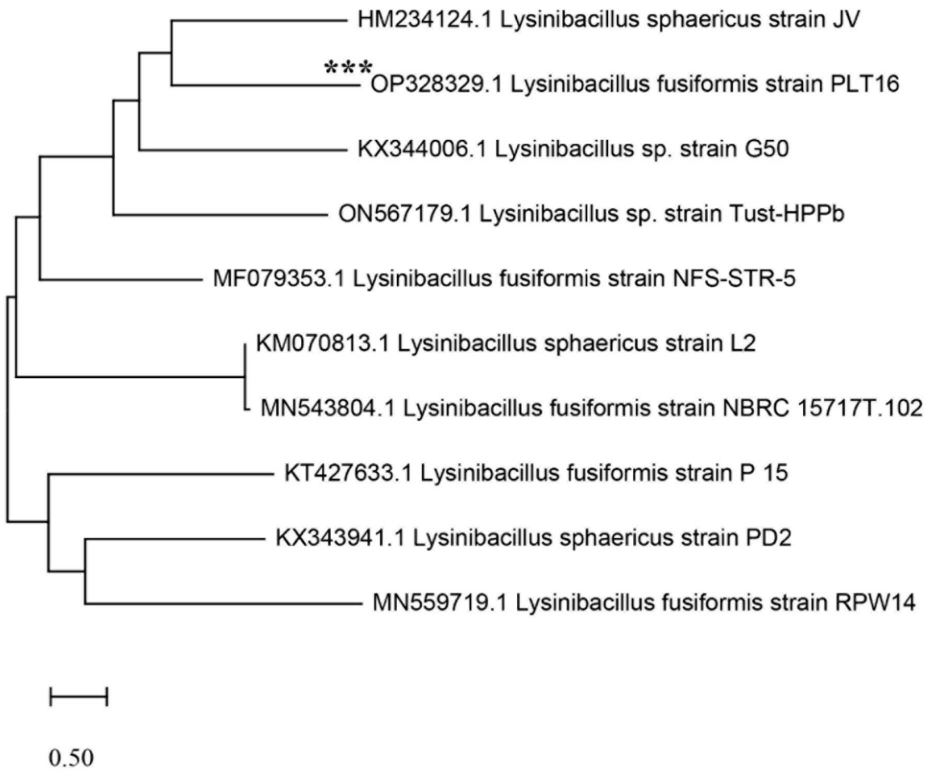

Figure S3. Phylogenetic tree of PLT16, which was constructed using 16S rRNA sequences by neighbor-joining (NJ) and maximum-likelihood methods.

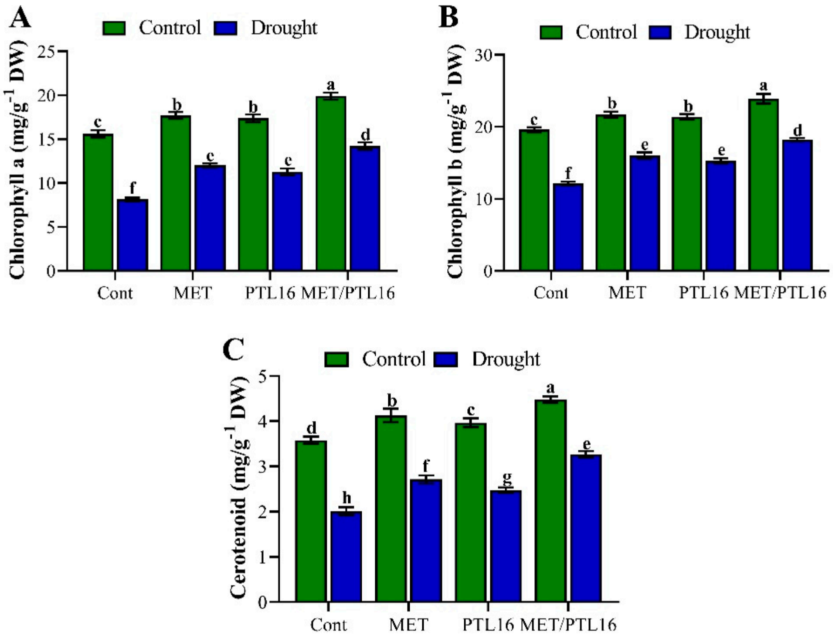

Figure S4. Effects of melatonin and isolate PLT16 application, on (A) chlorophyll *a*, (B) chlorophyll *b*, and (C) carotenoid content in soybean plants under drought stress. Each data point is the mean of three replicates. Error bars represent the standard error of the mean. Bars with different letters are significantly different from each other by Duncan's multiple range test at $p \leq 0.05$.

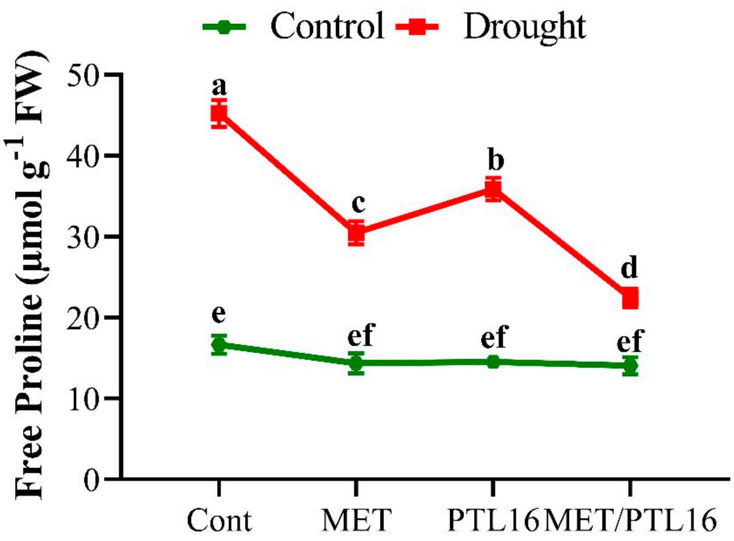

Figure S5. Effects of melatonin and isolate PLT16 application, on proline content in soybean plants under drought stress. Each data point is the mean of three replicates.

Error bars represent the standard error of the mean. Bars with different letters are significantly different from each other by Duncan's multiple range test at $p \leq 0.05$.

Table S1. GC/MS-SIM condition used for abscisic acid quantification.

Equipment	Hewlett-Packard 6890, 5973N Mass Selective Detector
Column	HP-1 capillary column (30 m×0.25 mm i.d. 0.25 μm film thickness) (J & W Scientific Co., Folsom, CA, USA)
Carrier gas	He (40 mL/min.); head pressure of 30 kPa
Source temperature	250°C
Oven conditions	60°C (2 min.) → 10°C/min → 140°C (3 min) → 3°C/min → 170°C → 15°C/min → 285°C (8 min)
Injector temperature	200°C
Ionizing voltage	70 ev

Table S2. GC/MS-SIM condition used for jasmonic acid (JA) quantification.

Equipment	Hewlett-Packard 6890, 5973N Mass Selective Detector
Column	HP-1 capillary column (30 m×0.25 mm i.d. 0.25 μm film thickness) (J & W Scientific Co., Folsom, CA, USA)
Carrier gas	He (40 mL/min.); head pressure of 30 kPa
Source temperature	250°C
Oven conditions	60°C (2 min.) → 10°C/min → 140°C (3 min) → 3°C/min → 170°C → 15°C/min → 285°C (8 min)
Injector temperature	200°C
Ionizing voltage	70 ev

Table S3. HPLC condition used for analysis and quantficantion of salicylic acid.

Equipment	Shimadzu LC-10
Column	HP hypersil ODS (particle size 5μm, pore size 120 A)

Wave length	Excitation 305 nm, Emission 365 nm
Detector	RF-10Axl (fluorescence detector)
Oven condition	60℃ (1min) → 15℃/min →200℃ →5℃/min →250℃ →10℃/min →280℃
Solvent A	100% MeOH
Solvent B	100% water in 0.5% acetic acid
Flow rate	1.0 ml/min

Table S4. List of gene and their primer used for real-time PCR analysis.

**Genes Name**		**Primers**
GmERD1	**F**	5′-CGTCCAGAATTGCTCAACAG-3′
	**R**	5′-TGGGGTTATAGCCTTGTTGG-3′
GmNCED3	F	5′- ACCACCTCTTCGACGGCGACGGAATGGT-3′
	R	5′- ATGGCGAGGAGTTTTCCGTTGAAGAAGA-3′
GmDREB2	**F**	5′-GTTTTGGAATTGAGACAGGC-3′
	**R**	5′-ACCAACCATTTGACATAACG-3′
**GmbZIP1**	**F**	5′- ACCAACACCAACAACATCCA-3′
	**R**	5′- TTGAATGCTCAGCAGCAACT-3′
ACT11	**F**	5′- CGGTGGTTCTATCTTGGCATC-3′
	**R**	5′- GTCTTTCGCTTCAATAACCCTA-3′
